# Comments: The doctor's dilemma: Truth telling

**DOI:** 10.4103/0019-5545.44912

**Published:** 2009

**Authors:** Amit Agrawal

**Affiliations:** Department of Surgery, Datta Meghe Institute of Medical Sciences, Sawangi (Meghe), Wardha, India. E-mail: dramitagrawal@gmail.com

Sir,

I read the article “The doctor's dilemma: Truth telling”[1] with great interest. The author has discussed the importance and need of truth telling with a well quoted contemporary example with an excellent explanation. At the same time it emphasizes that there was an era when facilities were meager and the information was not readily accessible. The truth and trust were strong instruments in the management of the patient. The advancements in the field of health care and availability of this knowledge mainly because of internet access have changed the circumstances and these developments probably have generated the need to redefine the values. In many circumstances people may have enough information (though generalized) about the health problems and it increases the responsibility of the managing physician while providing the details for an individual. It is the time whether these details should we called as truth, facts or available evidence. I agree with the author[1] that long-term relationships between the physician and the patient can be strengthened by revealing the truth and providing the valid information definitely at all the time. This holds true even for many short-term relationship circumstances particularly in era where the development in medical field has increased the longevity with persisting morbidity (i.e. congenital anomalies). The truth should be reveled in his entirety[1] and we can take this as an opportunity and accept as a challenge to find out how (what) should we call it *(truth, facts* or *available evidence)*, how (what) to tell to the patient, how (what) we should tell to the caregivers, the time, the extent and in how many steps we should reveal it.
